# Uncovering the effects of grazing exclusion duration on plant richness and biomass in alpine grasslands using the price equation

**DOI:** 10.3389/fpls.2026.1762528

**Published:** 2026-01-30

**Authors:** Xin Pu, Tan Feng, Lei Sun

**Affiliations:** 1 Xizang Agricultural and Animal Husbandry University, Nyingchi, China; 2 Qiangtang Alpine Grassland Ecosystem Research Station, Nagqu, China

**Keywords:** alpine grasslands, biomass redistribution, enclosure, price equation, species turnover

## Abstract

Grazing exclusion is a key strategy for restoring degraded alpine grasslands. However, the mechanisms underlying plant species richness and biomass responses to long-term enclosure, particularly species turnover and biomass redistribution, remain unclear. This study compared plant community composition, diversity, and biomass across alpine grasslands on the Qinghai–Tibet Plateau enclosure for 2, 6, 13, and 18 years and free-grazing sites. The Price equation was used to quantitatively partition the independent contributions of lost, gained, and persisting species to changes in diversity and biomass, revealing how the long-term grazing exclusion affects biodiversity and biomass. After 2 years of enclosure, both diversity and biomass increased simultaneously. The biomass increase was primarily derived from the increased biomass of persisting species, while newly gained species contributed little. After 6 years, biomass reached peak value due to further increases in the biomass of persisting gramineous species. However, the loss of weedy species and reduction in species gains caused a diversity decline. After 13 and 18 years of enclosure, the biomass of persisting species began to decline, leading to a gradual decrease in total biomass. In summary, short-term enclosure should prioritize the recovery and conservation of native species rather than the colonization by new species. During the mid-term, attention should be paid to the potential negative impact of overgrown native gramineous species on overall diversity. Long-term grazing exclusion should be avoided where possible to prevent ecosystem degradation. This study provides a novel paradigm for alpine grassland restoration by disentangling community-level dynamic processes.

## 1 Introduction

As the world’s largest high-altitude grassland ecosystem, the alpine grasslands of the Qinghai–Tibet Plateau play an irreplaceable role in maintaining water cycles, carbon balance, regional biodiversity, and the sustainable supply of pastoral resources (Wei et al., 2012; Zhu et al., 2023). However, human activities such as overgrazing, has caused significant degradation across the region (Mcsherry and Ritchie, 2013; Li et al., 2017). This is mainly manifested by the continuous decline of aboveground biomass and substantial losses in plant diversity (Chaplin-Kramer et al., 2019; Bardgett et al., 2021; Wang et al., 2022). Grazing exclusion has been widely implemented as a restoration measure. Although it can promote rapid vegetation recovery in the short term, its long-term effects show comprehensive spatial and temporal heterogeneity (Zhu et al., 2016; Li et al., 2018; Qin et al., 2025). Notably, plant diversity and aboveground biomass respond differently to varying durations of enclosure and sometimes display stage-specific antagonism (Wu et al., 2009; Zhang et al., 2015). However, the regulatory mechanisms of the dynamic responses remain unclear and have become a major scientific bottleneck, restricting the effective design and adaptive management of restoration strategies.

**Figure 1:**
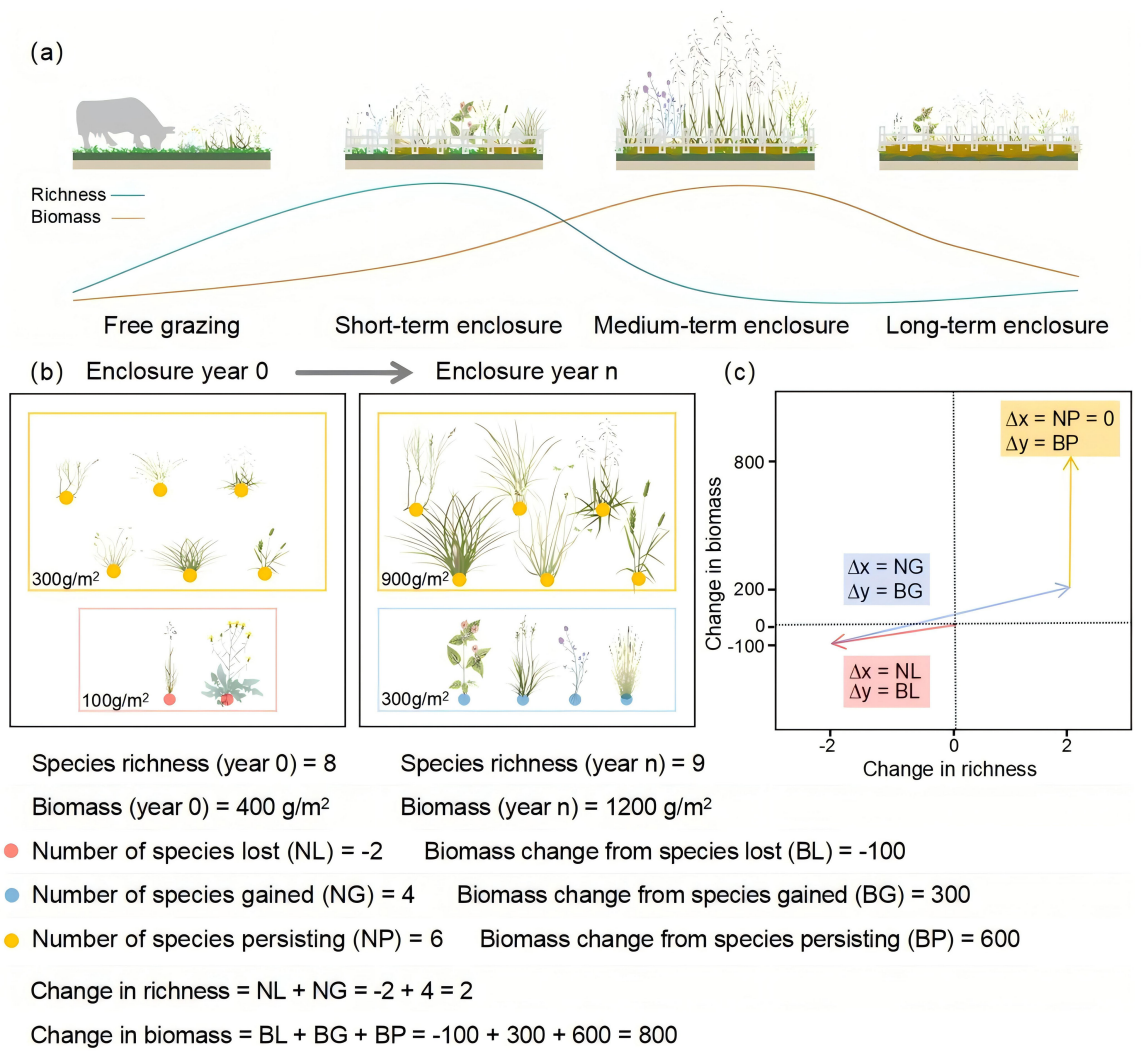
**(a)** Overall patterns of species richness and aboveground biomass under different enclosure durations; **(b)** Comparison of community trophic structure before (Year 0) and after (Year n) of enclosure using the modified Price equation proposed by Bannar-Martin et al. (2018), distinguishing species dynamics by red (lost species), blue (gained species), and orange (persisting species), and analyzing the additive effects of each component on species richness and aboveground biomass change; **(c)** Changes in community species richness and aboveground biomass, with red indicating lost species, blue for gained species, and orange for persistent species. Plant materials from https://ian.umces.edu/work-with-us/.

Short-term grazing exclusion significantly promotes the recovery of native vegetation and the colonization of new species by removing grazing pressure, leading to simultaneous increases in plant diversity and aboveground biomass (Li et al., 2017; Zhan et al., 2022; Yu et al., 2024). However, as the duration of enclosure extends, species with high resource-capture efficiency rapidly occupy ecological niches and accumulate aboveground biomass, gaining a strong competitive advantage and suppressing the survival of others, which might result in an imbalanced state where high biomass coexists with low diversity (Sun et al., 2020; Zhang et al., 2021; Campana et al., 2024). Meanwhile, long-term enclosure leads to numerous litter accumulation, which suppresses seed input, hinders seedling establishment, and limits nutrient cycling, which significantly limits species growth and new species colonization, resulting in a decline in both plant diversity and aboveground biomass (Ruprecht and Szabo, 2012; Xiong et al., 2014; Chen et al., 2018; Zhang et al., 2019) (**Figure 1a**). The basis of these stage-specific response patterns stem from the interactive processes of species turnover (loss, gain, and persistence dynamics) and redistribution of aboveground biomass (Zhao et al., 2022; Avolio et al., 2021). However, conventional research paradigms are limited to static community-level metrics (such as total biomass and plant diversity), with few studies quantitatively assessing the underlying mechanisms of the species turnover and biomass redistribution in shaping ecosystem function (Yao et al., 2019; Zhao et al., 2022). For example, is the increase in aboveground biomass under short-term enclosure driven by the regenerative capacity of persisting species, or is it due to functional contributions from newly colonized species? In the mid-term enclosure, are the dominant species in biomass derived from the original community or from the newly introduced components? Explaining these mechanisms requires a dynamic model based on community reassembly processes, enabling precise quantification of the ecological effects of different community components (Leibold et al., 2017; Ulrich et al., 2022).

In recent years, the Price equation has been used as a key tool for analyzing community dynamics and has played an important role in studies exploring the effect of climate change and human activities biodiversity and ecosystem functioning (Bannar-Martin et al., 2018; Ladouceur et al., 2022). Based on the Price equation framework, this study quantified the plant community reassembly process into three components: lost species, gained species, and persisting species (**Figure 1b**). Changes in plant diversity are determined by the number of gained and lost species, while total aboveground biomass is driven by the additive contributions of all three components: lost, gained, and persisting species (Leibold et al., 2017; Bannar-Martin et al., 2018) (**Figure 1c**). If the increase in aboveground biomass during short-term enclosure primarily results from the growth of already dominant species, it suggests the recovery of total biomass is more reliant on the ecological resilience of persisting species than on colonization by new species or increased plant diversity (Zhang et al., 2025). Meanwhile, changes in plant diversity are determined solely by species loss and gain and are independent of changes in persisting species. Thus, when variations in the biomass of persisting species play a decisive role in total biomass change, plant diversity and aboveground biomass may become decoupled (Li et al., 2018; Ulrich et al., 2022). This analytical framework reveals the underlying mechanisms of plant diversity and aboveground biomass responses in alpine grasslands to varying durations of grazing exclusion by quantifying the independent effects of species turnover components and biomass redistribution.

Focusing on the alpine grasslands of the Qinghai-Tibetan Plateau, this study systematically surveyed plant community composition and aboveground biomass across four grazing exclusion sites (enclosed for 2, 6, 13, and 18 years) and one freely grazed control site. Using the Price equation as an analytical framework, this study specifically examined the following: (1) the patterns of stage-specific responses in plant diversity and aboveground biomass to varying enclosure durations in degraded alpine grasslands; (2) the contribution mechanisms of species loss, gain, and persistence to changes in plant diversity and aboveground biomass under different enclosure durations. By deconstructing the roles of individual components in community turnover, this study clarifies the response thresholds and driving pathways of plant diversity and aboveground biomass to enclosure duration in degraded alpine grassland ecosystems. These findings aim to provide theoretical support for optimizing adaptive management strategies in alpine grasslands and offer quantitative evidence for formulating differentiated grazing prohibition policies.

## 2 Materials and methods

### 2.1 Study area introduction

The study was conducted in Bangor County, located in the central-northern core area of the Qinghai-Tibetan Plateau (29°55′–32°15′N, 88°56′–91°18′E), with an average elevation of 4,700 meters. The region experiences a plateau subalpine semi-arid monsoon climate characterized by long, cold winters and short, cool summers. There is no absolute frost-free period throughout the year. The annual mean temperature is -1.2°C, with the warmest month (July) averaging below 14°C and the coldest month (January) averaging below -10°C. Annual precipitation ranges from 289 to 390 mm, with 60–80% concentrated during the plant growing season (June to August). The local alpine grassland is dominated by *Stipa purpurea*, with associated species including *Heteropappus bowerii*, *Festuca coelestis*, and *Carex moorcroftii*.

### 2.2 Experimental design and sampling

The study was conducted in the surrounding area of the Qiangtang Alpine Grassland Ecosystem Research Station (31°34′12″N, 89°52′12″E, 4,634 m a.s.l.; **Supplementary Figure S1**). Based on a gradient of enclosure duration, we selected four recovery sites (established in 2006, 2011, 2018, and 2022, representing 18, 13, 6, and 2 years of enclosure, respectively) along with one freely grazed site maintained under traditional nomadic practices. To minimize site-to-site interference, the distance between sampling sites was kept ≥ 20 km. All sites had similar vegetation composition prior to treatment. The enclosed sites were strictly protected from grazing using 1.5-meter-high wire fencing.

Field surveys were conducted in late August 2024, during the peak of the growing season. At each site, we established a transect at a relatively flat central location. Along each transect, five 100 m² (10 m × 10 m) sampling units were set at 100-meter intervals. Within each unit, five 0.5 m × 0.5 m quadrats were placed in a W-shaped pattern, the morphological traits and identity of all plant species in each quadrat were recorded. After *in-situ* observations, all aboveground parts of plants within each quadrat were harvested by clipping at ground level, and stored in labeled envelopes sorted by species. In the laboratory, plant samples were oven-dried at 65°C to a constant weight to determine aboveground biomass for each species.

### 2.3 Analysis methods

We analyzed changes in plant species richness and aboveground biomass using the Price equation framework (Bannar-Martin et al., 2018). Employing a chronosequence approach, we independently compared each enclosure site (2, 6, 13, and 18 years) against the same continuously grazed control site as the baseline (**Figures 1b, c**). The net biomass change was partitioned into three components: the biomass of species lost (species present in the freely grazed site only), the biomass of species gained (species present in the enclosure site only), and the biomass change of species that persisted (species present in both). To assess the effects of enclosure duration on community dynamics, the Analysis of variance (ANOVA) was conducted on the following variables: species richness, total aboveground biomass, and the number and aboveground biomass of lost, gained, and persistent species. The Tukey’s HSD (Tukey) test was used for *post hoc* comparisons, and significant letters were marked to clarify the significance of the differences between the factors. Based on the method of Bannar-Martin et al. (2018), the species richness and aboveground biomass changes of different community components deconstructed by the Price equation were plotted to directly display the community change characteristics of each component. NMDS analysis was used to compare the differences in community composition among different enclosure durations. General linear models (GLMs) were used to assess correlations between species richness and the numbers of lost and gained species, as well as between total aboveground biomass and aboveground biomass of lost, gained, and persistent species. Prior to ANOVA and GLMs, the normality of residuals and homoscedasticity of variances were verified using Shapiro-Wilk and Levene’s tests (*P* > 0.05), respectively. All analyses were conducted using R version 4.0.

## 3 Results

### 3.1 Responses of species richness and aboveground biomass to enclosure duration

**Figure 2:**
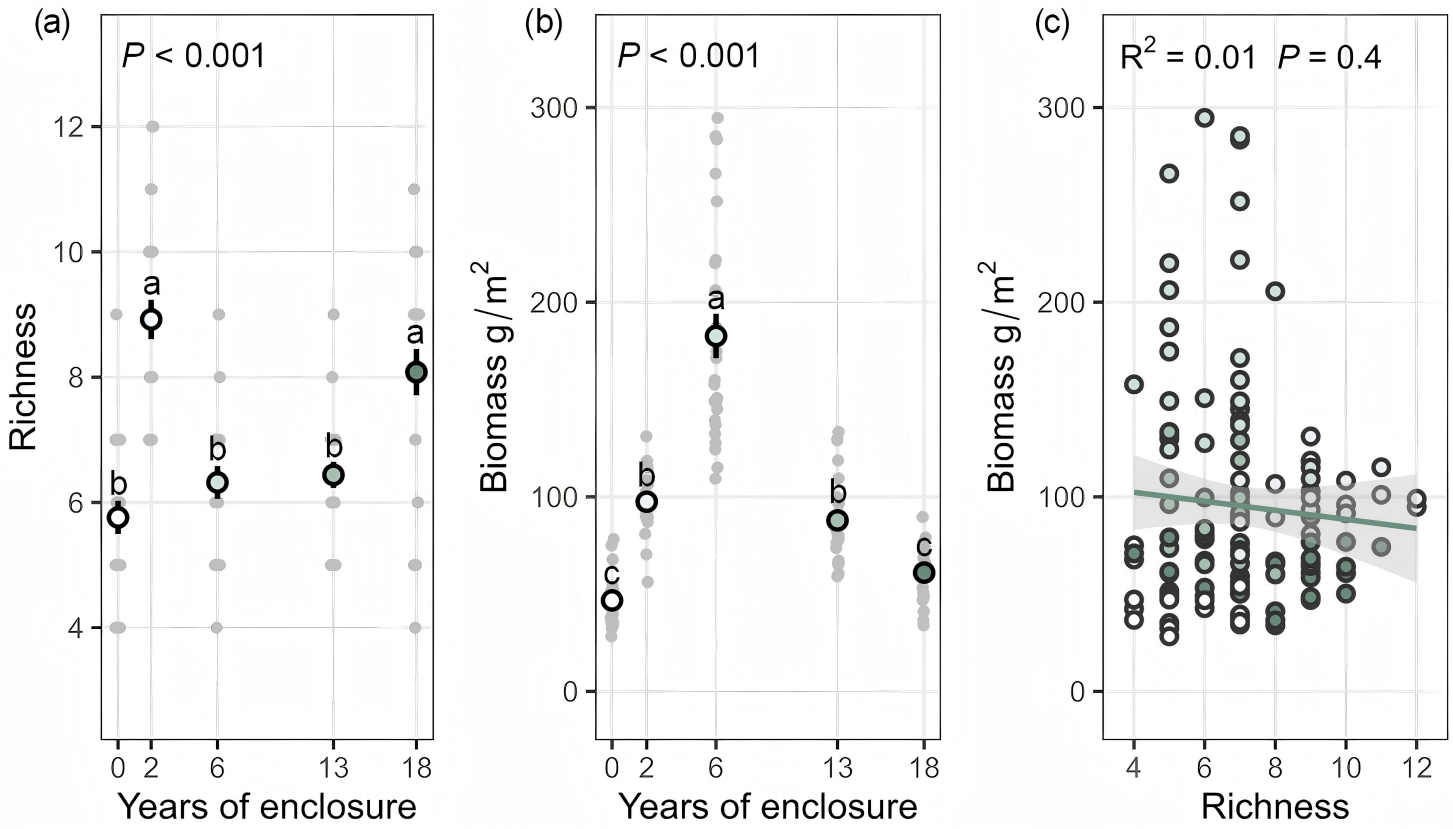
Effects of enclosure duration on plant species richness **(a)** and aboveground biomass **(b)** (mean ± SE; different letters indicate significant differences at *P* < 0.05); Relationship between species richness and aboveground biomass **(c)**. Additional statistical metrics are provided in **Supplementary Table S1**.

After 2 years of enclosure, both species richness and aboveground biomass increased significantly (species richness: +54.86%; aboveground biomass: +108.78%, *P* < 0.05, Tukey; **Figure 2a**). After 6 years of enclosure, the two indicators diverged: species richness declined significantly (-29.15%, *P* < 0.05, Tukey), while aboveground biomass continued to rise and reached its peak (+291.86% compared to the grazed control, *P* < 0.05, Tukey). After 13 years of enclosure, species richness showed no significant change, but aboveground biomass decreased sharply (-52.02%, *P* < 0.05, Tukey). At 18 years, species richness began to recover moderately (+25.47%), whereas aboveground biomass continued to decline significantly (-30.52%, *P* < 0.05, Tukey; **Figure 2b**). Overall, there is no correlation between plant species richness and aboveground biomass (**Figure 2c**). Additionally, NMDS results indicated that species composition became increasingly differentiated with longer enclosure durations (**Supplementary Figure S2**).

### 3.2 Price equation analysis of species richness and aboveground biomass change sources

**Figure 3:**
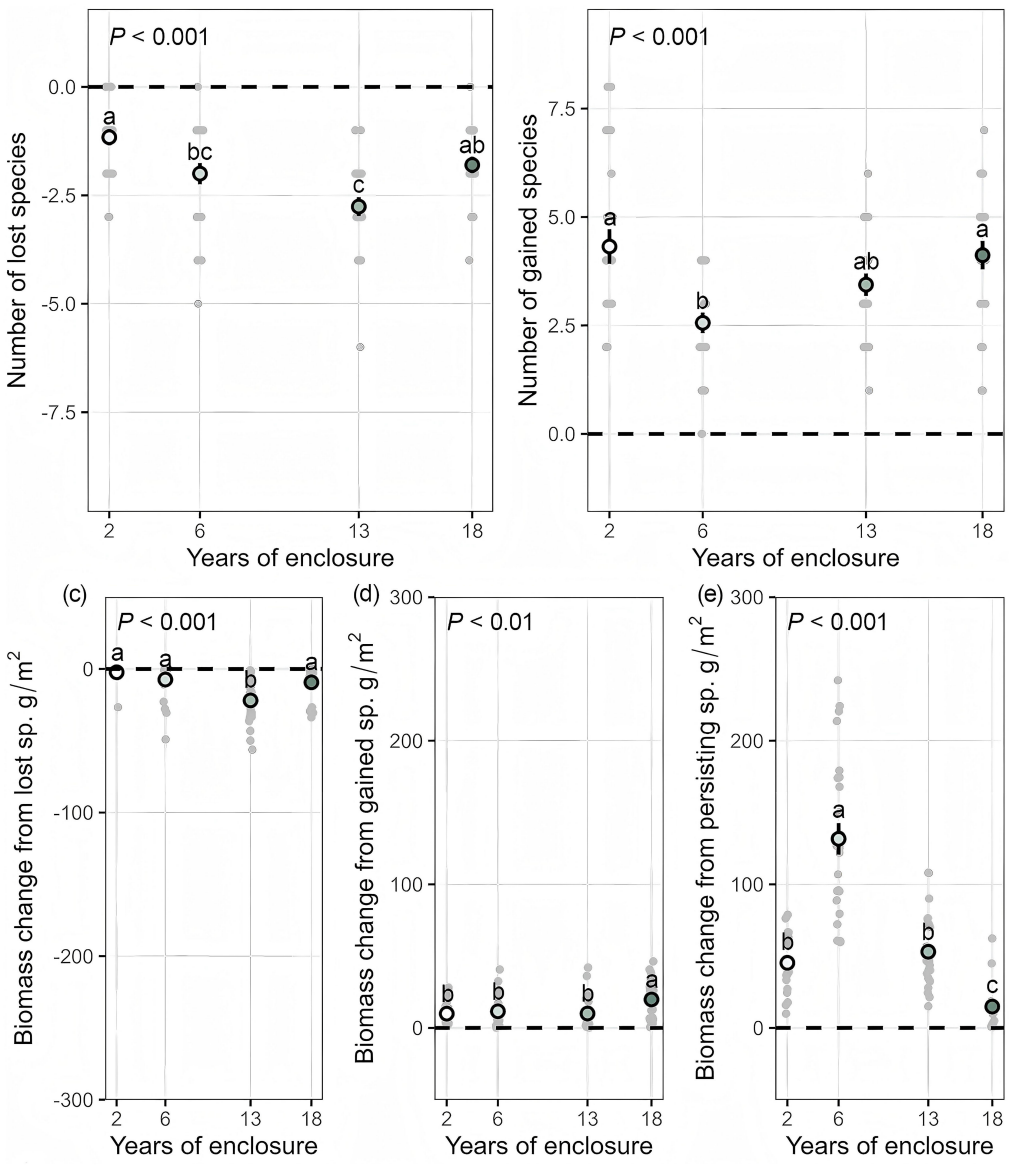
Dynamics of species lost **(a)** and gained **(b)**, and the effects of lost **(c)**, gained **(d)**, and persisting **(e)** species on aboveground biomass (mean ± SE; different letters indicate significant differences at *P* < 0.05). Additional statistical metrics are provided in **Supplementary Table S1**.

The number of lost species gradually increased during the 2, 6, and 13 years of enclosure (2 years: 1.16 species; 6 years: 2 species; 13 years: 2.76 species) and declined in the 18th year (1.8 species; **Figure 3b**). The number of gained species peaked at 2 years (4.32 species), declined at 6 years (2.56 species), and then gradually increased through 13 and 18 years (13 years: 3.44 species; 18 years: 4.12 species; **Figure 3a**).

The negative effect of species loss on total aboveground biomass intensified over the 2, 6, and 13-year enclosure durations (2 years: -2.32 g; 6 years: -7.39 g; 13 years: -21.9 g) but decreased in the 18th year (-9.32; **Figure 3c**). The contribution of gained species to total aboveground biomass remained relatively stable across the first three stages (2–13 years: 9.92–11.6 g), with a significant increase observed in the 18th year (19.8 g, *P* < 0.05, Tukey; **Figure 3d**). The biomass of persisting species initially increased, peaking at 6 years (132 g), but then significantly declined in both the 13th and 18th years (13 years: 53 g; 18 years: 3.82 g, *P* < 0.05, Tuke; **Figure 3e**). Notably, the variation in the proportion of aboveground biomass of the persisting species, *Stipa purpurea*, followed the same trend observed in persisting species, reaching its peak of 83.54% in the 6th year followed by a gradual decline (**Supplementary Figure S3**).

**Figure 4:**
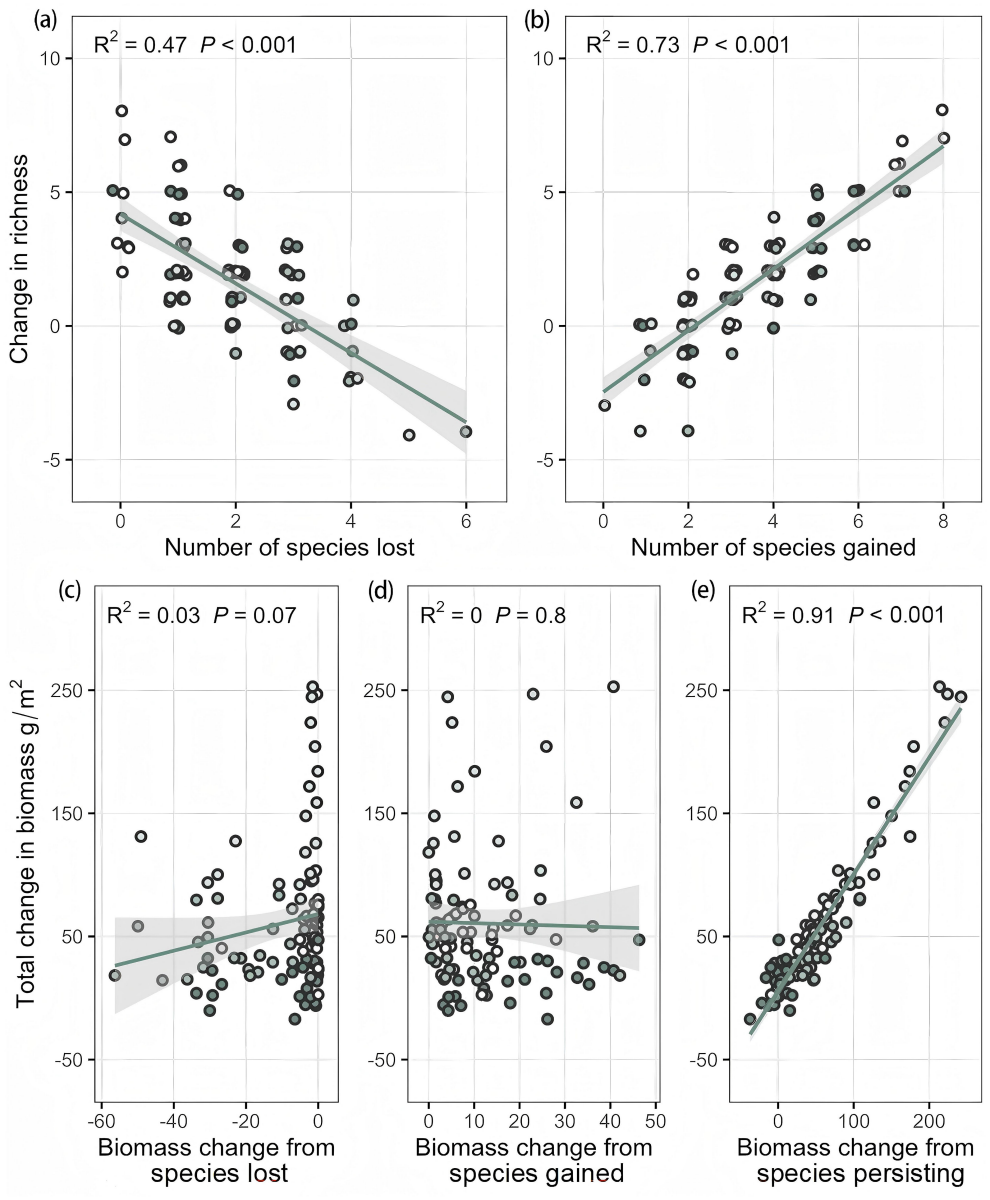
Correlation analysis between plant species richness and lost **(a)** and gained **(b)** species, and between lost **(c)**, gained **(d)**, and persisting **(e)** species and aboveground biomass.

Price equation analysis indicated that the number of species gained was consistently higher than species lost throughout the 2–18-year enclosure period (**Supplementary Figure S4**), resulting in an overall increase in species richness compared to the grazed control. Moreover, correlation analysis revealed that changes in species richness were more strongly associated with the changes in gained species. As expected, species richness was significantly negatively correlated with the number of lost species (R² = 0.47, *P* < 0.001, GLMs; **Figure 4a**), and extremely significantly positively correlated with the number of gained species (R² = 0.73, *P* < 0.001, GLMs; **Figure 4b**). Changes in total aboveground biomass were primarily driven by persisting species. In the 2, 6, and 13 years of enclosure, the contribution of persisting species to total aboveground biomass was much greater than the contribution of lost and gained species. This contribution consistently increased during the 2nd and 6th years and then consistently declined in the 13th and 18th years. Correlation analysis further showed that changes in total aboveground biomass were significantly positively correlated with changes in the biomass of persisting species (**Figure 4e**), but were not correlated with the biomass of lost (**Figure 4c**) or gained species (**Figure 4d**). Additionally, the results indicated that persistent species were primarily gramineous species (**Supplementary Figure S5**), while both lost and gained species were primarily forbs (**Supplementary Figure S6**). Furthermore, changes in total aboveground biomass were driven by the biomass fluctuations of persisting gramineous species, whereas changes in species richness were mainly driven by fluctuations in the number of forb species.

## 4 Discussion

Short-term enclosure (2 years) significantly enhanced both species richness and aboveground biomass, displaying a typical co-recovery pattern of species richness and biomass. This finding aligns with global trends observed during the short-term restoration of degraded grasslands (Pei et al., 2008; Mcsherry and Ritchie, 2013; Jing et al., 2014). This may be due to soil compaction, nutrient loss, and grazing pressure caused by overgrazing being rapidly relieved after grazing prohibition, promoting the regeneration of persisting species and the colonization of gained species (Golodets et al., 2010; Tang et al., 2016). Price equation analysis revealed that, although total community aboveground biomass increased, this increase was primarily driven by the biomass accumulation of persisting native species, with a relatively minor contribution from newly gained species (**Figure 3d**). These persisting native species well adapted to harsh environments are mainly perennial clonal plants (such as *Stipa purpurea*), they have stronger resilience and recovery potential, once environmental stress was removed, they rapidly restored their aboveground biomass and population size through clonal propagation (D’Hertefeldt et al., 2011; Wang et al., 2021). These results indicate that the restoration efficiency of short-term enclosure depends on the ecological resilience of functional groups of persisting species rather than on the colonization of gained species. Therefore, in management practices, priority should be given to the conservation of native functional groups rather than relying on the establishment of newly gained species.

In the mid-term enclosure period (6 years), aboveground biomass reached the peak (**Figure 2b**), while species richness significantly declined (**Figure 2a**). Notably, the persisting gramineous species (*Stipa purpurea*) accounted for 83.54% of the biomass at this stage (**Supplementary Figure S3**), quantitatively demonstrating its role in resource monopolization. This non-equilibrium ecosystem pattern characterized by “low species richness and high aboveground biomass” is primarily the result of high resource-capture efficiency species (particularly gramineous species) rapidly occupying multiple ecological niches through phenotypic plasticity (**Supplementary Figure S5**). These species expand quickly via clonal propagation and root extension, forming dense canopies that exert strong competitive exclusion, suppressing the growth of native species and the establishment of new species, ultimately simplifying community composition (Hanslin et al., 2019; Viana et al., 2022). This pattern of resource monopolization aligns with findings from studies on the Eurasian steppe and North American tallgrass prairie, where high resource-capture efficiency species drive community simplification by occupying spatial niches (Yu et al., 2025). Price equation analysis further revealed that the dominant contributors to aboveground biomass during the mid-term enclosure were persisting gramineous species (e.g., *Stipa purpurea*) and that their niche preemption and competitive advantages were the primary drivers of changes in persisting species biomass (Hanslin et al., 2019; Song et al., 2025). This mechanism is consistent with analyses of global grasslands​ using the Price equation framework, which partition biodiversity effects and reveal that biomass increases under resource enrichment are predominantly driven by the growth of persistent species rather than species turnover (Ladouceur et al., 2022). In addition, the decline in species richness in our study was due to the loss of forb species and the reduction in gained species. However, both lost and gained species had relatively minor impacts on total aboveground biomass, which explains the asynchronous response between diversity and biomass (Ladouceur et al., 2022; Campana et al., 2024; Song et al., 2025). Therefore, during mid-term enclosure, moderate grazing or mowing should be introduced to mitigate the negative impacts of excessive growth of persisting native species, particularly gramineous species, on species richness.

Long-term enclosure (13 years) resulted in a dual decline of species richness and aboveground biomass, which is consistent with the widespread self-inhibitory effects observed in long-term enclosure of degraded grasslands globally (Sun et al., 2020; Song et al., 2020; Jiang et al., 2023). Grazing exclusion protects the tall species while limiting light availability under their canopies, which in turn suppresses the growth and diversity of short species (Zhang et al., 2019; Song et al., 2025). Meanwhile, in the late stages of enclosure, numerous withering accumulates and inhibits seed input and seedling growth through multiple mechanisms, including physical obstruction, allelopathic chemical release, altered interspecific competition, and disrupted nutrient cycling, ultimately limiting native plant growth and new species colonization (Hobbie et al., 2015; Johnson et al., 2018; Zhan et al., 2024). Notably, the rebound in species richness observed in year 18 was driven by an increase in the number of gained forb species, However, although these species have functional compensation capacity, they failed to reverse the declining trend in aboveground biomass. Therefore, long-term enclosure should be avoided where possible, or targeted interventions, such as withering removal or moderate grazing, should be implemented to break the self-inhibitory state of the ecosystem (Jessen et al., 2023).

While this study provides novel insights into enclosure effects by quantifying community components through the Price equation, several limitations should be acknowledged. First, the chronosequence approach implies a space-for-time substitution, which may not fully account for inherent spatial variability among sites (e.g., soil properties or microtopography), despite our careful selection of sites with similar initial conditions. Long-term fixed-point monitoring would better verify these temporal causal mechanisms. Second, our interpretations of the underlying mechanisms—such as light competition, litter accumulation, and nutrient limitation—and the role of functional dominance are inferred from biomass dynamics and taxonomic identity rather than direct measurements of abiotic factors or specific plant functional traits (e.g., plant height, specific leaf area, leaf dry matter content, clonal traits). Consequently, the specific physiological drivers of the observed self-inhibitory effects remain hypothetical and require verification through trait-based analyses. Finally, the proposed management strategies remain conceptual. Future studies should determine specific operational parameters (e.g., grazing intensity and timing) through targeted experiments.

## 5 Conclusion

This study elucidates that the recovery trajectories of species richness and aboveground biomass during grassland enclosure are temporally asynchronous. While short-term exclusion fosters rapid recovery through the resurgence of persisting native species, prolonged enclosure shifts community dynamics from restoration to competitive exclusion, where excessive biomass accumulation of persistent dominant species eventually compromises diversity. Specifically, our chronosequence data indicates that the mid-term stage (approximately 6 years) represents a critical temporal threshold where biomass peaks while diversity significantly declines. Consequently, static enclosure should not be viewed as a universally applicable prescription. Instead, we propose a stage-dependent conceptual optimization framework. Management should transition from strict protection in the early phase—prioritizing the conservation of the native species pool—to active interventions in the mid-term (around year 6) to mitigate the exclusion of other species by persisting dominant species. While this study identifies the timing for intervention, future experiments are required to quantify specific operational parameters, such as optimal grazing intensities.

## Data Availability

The original contributions presented in the study are included in the article/**Supplementary Material**. Further inquiries can be directed to the corresponding author.
