# Supplementary material for: Uncovering the effects of grazing exclusion duration on plant richness and biomass in alpine grasslands using the price equation
Source: Front Plant Sci. 2026 Jan 30;17:1762528. doi: 10.3389/fpls.2026.1762528 (PMC12901424; doi:10.3389/fpls.2026.1762528)
Supplement: Supplementary file 1 [file Supplementaryfile1.docx]

Table S1 Results of one-way ANOVA to assess the effects of different grazing exclusion (2, 6, 13, and 18 years since grazing exclusion) on species richness, gained species numbers, lose species numbers, biomass, gained species biomass, lose species biomass and persisting species biomass. Bold values indicate statistical significance at the *P* < 0.05 level.

| Variables | Inoculum | | |
| --- | --- | --- | --- |
|  | F | *P* | Df |
| Species richness | 21.18 | **<0.001** | 4 |
| Gained species numbers | 6.47 | **<0.001** | 3 |
| Lose species numbers | 9.663 | **<0.001** | 3 |
| Biomass | 80.99 | **<0.001** | 4 |
| Gained species biomass | 4.714 | **0.00411** | 3 |
| Lose species biomass | 12.52 | **<0.001** | 3 |
| Persisting species biomass | 64.38 | **<0.001** | 3 |


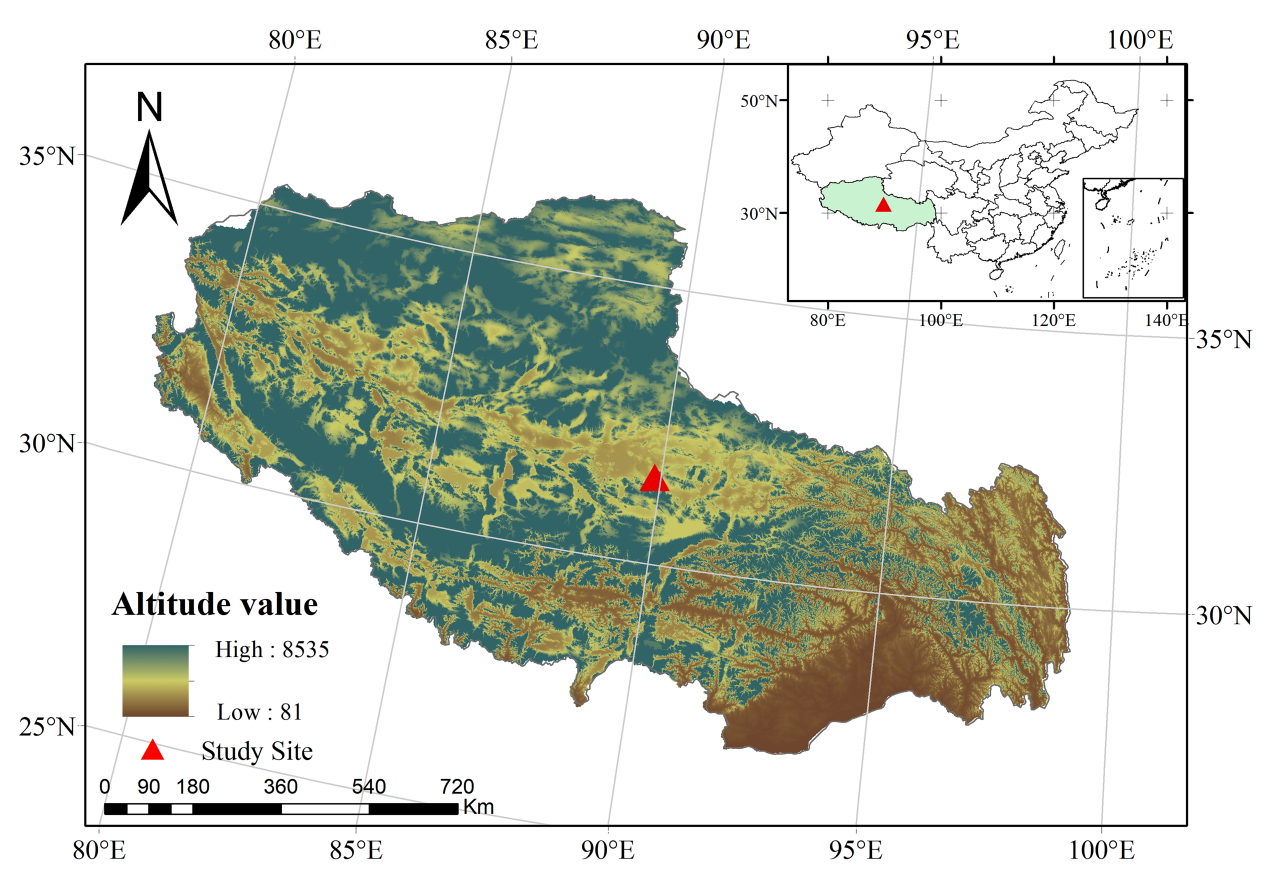


Figure S1 Map of the study area's geographical coordinates.


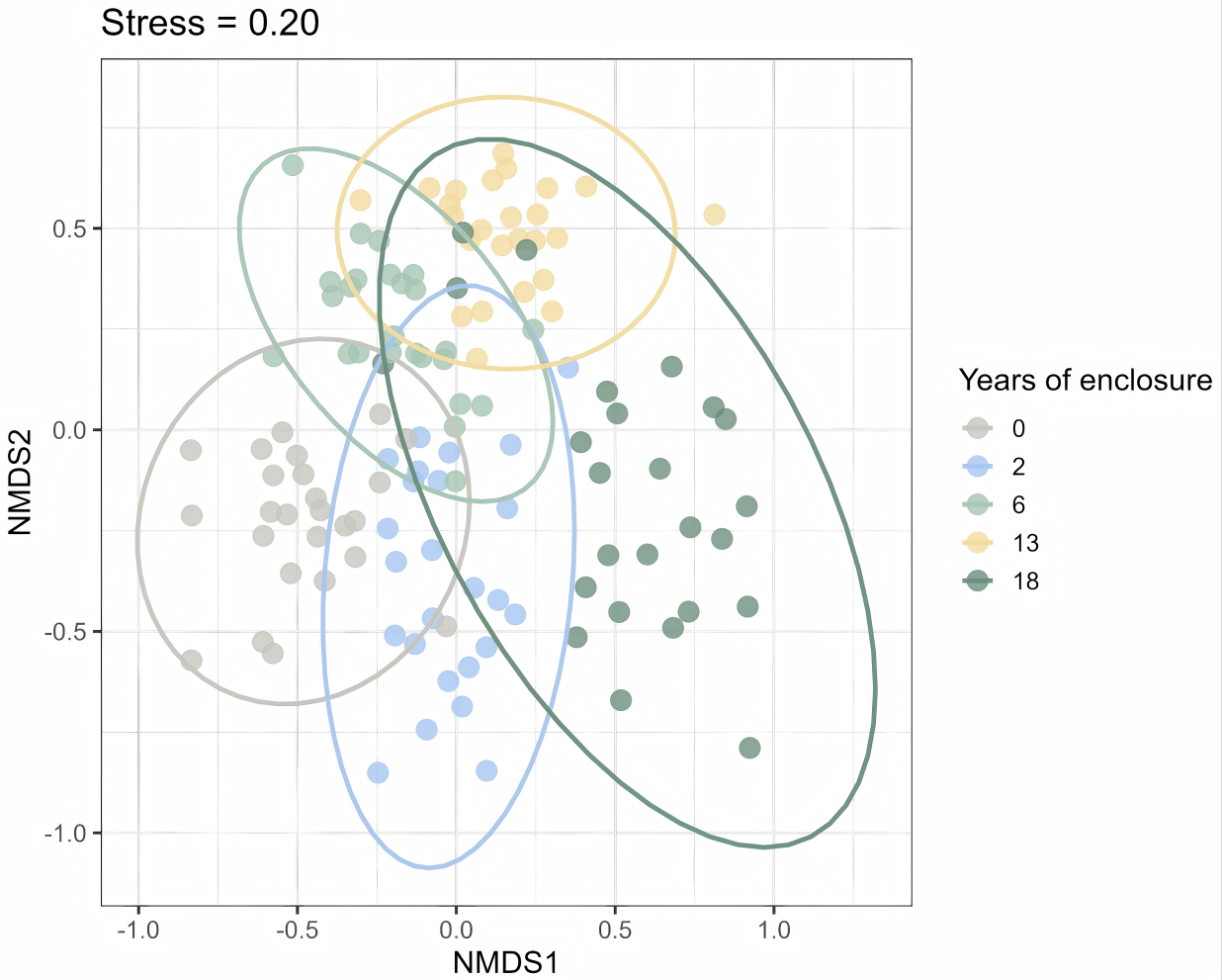
 Figure S2 NMDS analysis of species composition under different enclosure durations.


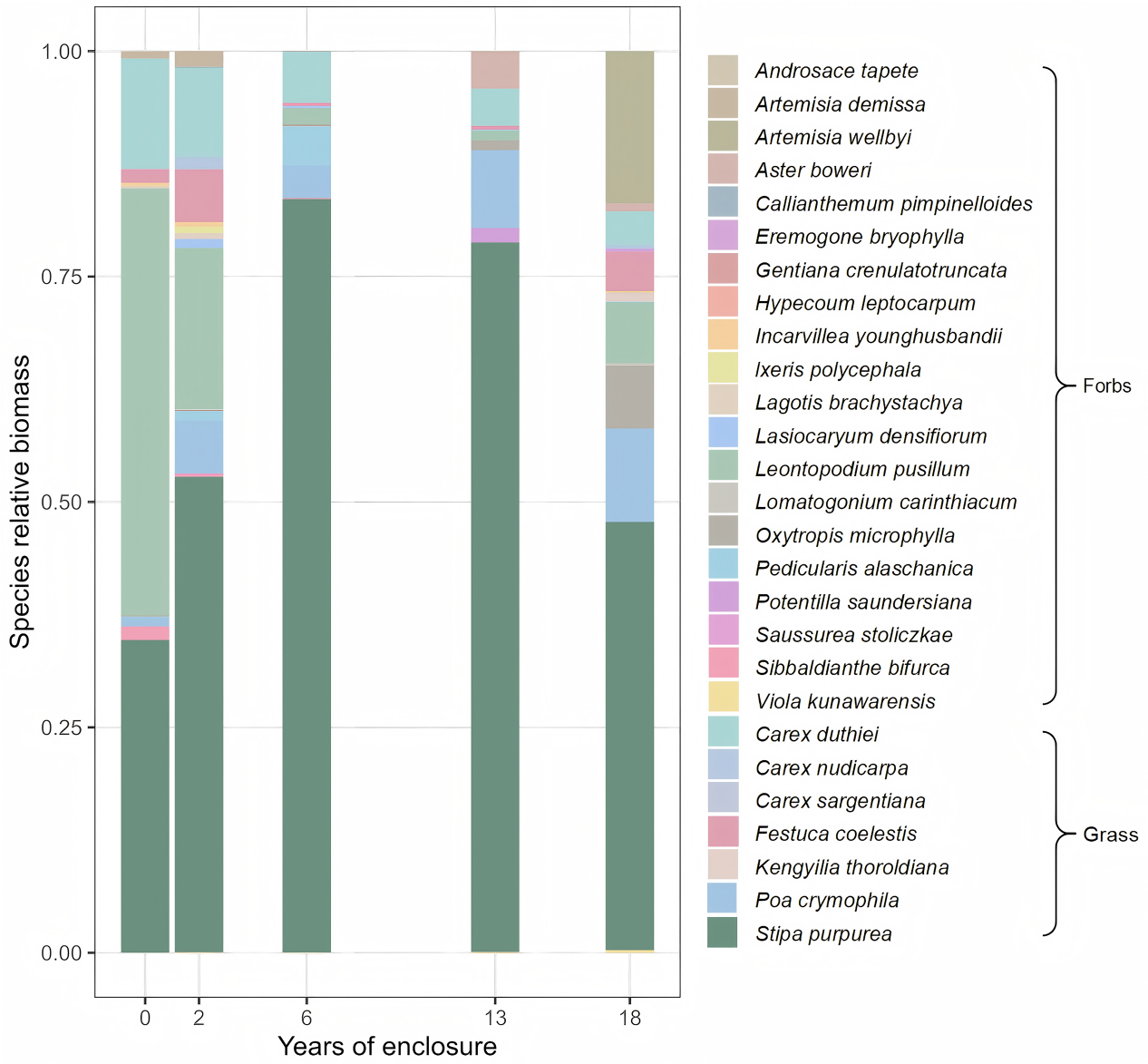


Figure S3 Stacked percentage chart of aboveground biomass composition by species under different enclosure durations.

Figure S4 Changes in species richness and aboveground biomass under different enclosure durations.


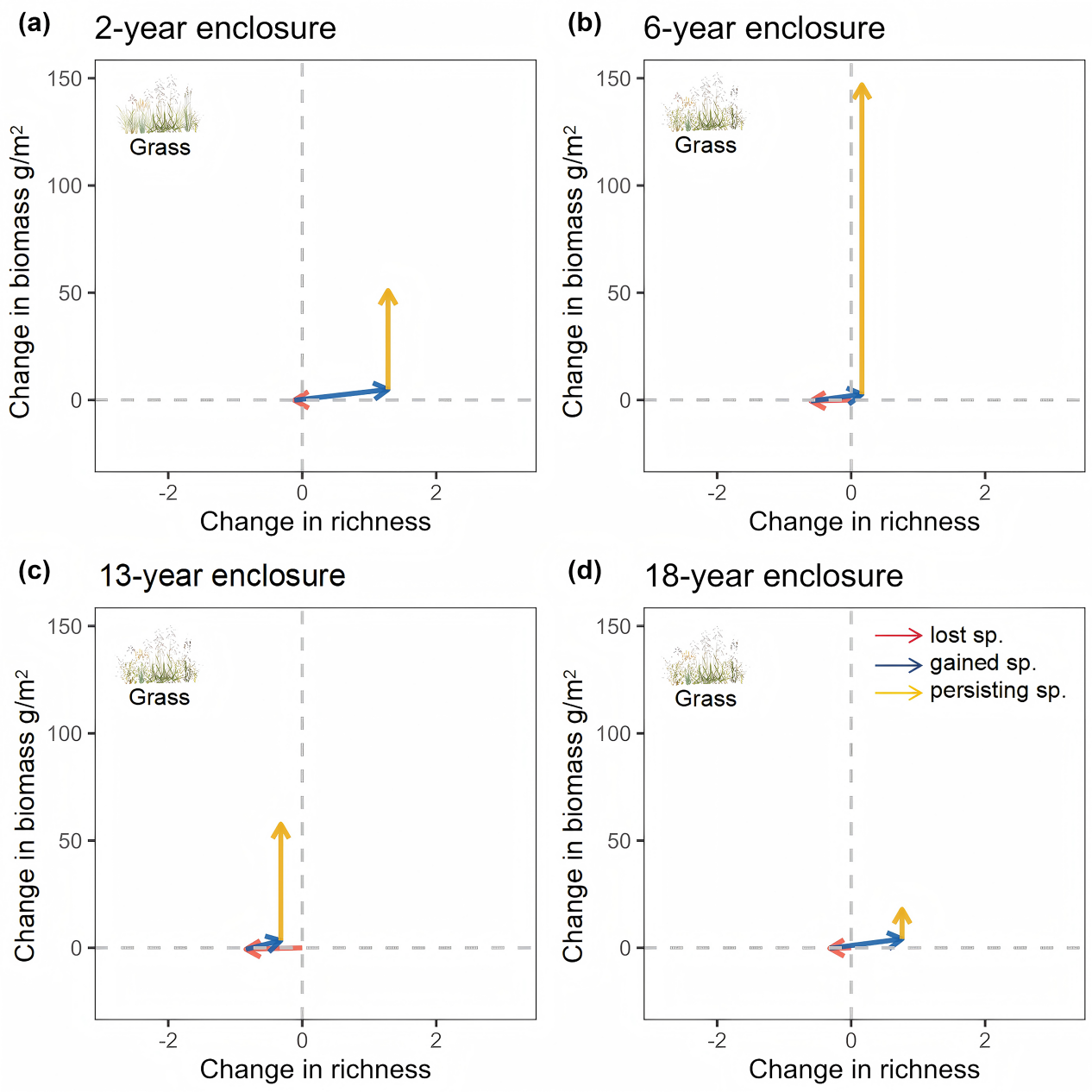


Figure S5 Changes in diversity and aboveground biomass of Grass species under different enclosure durations.


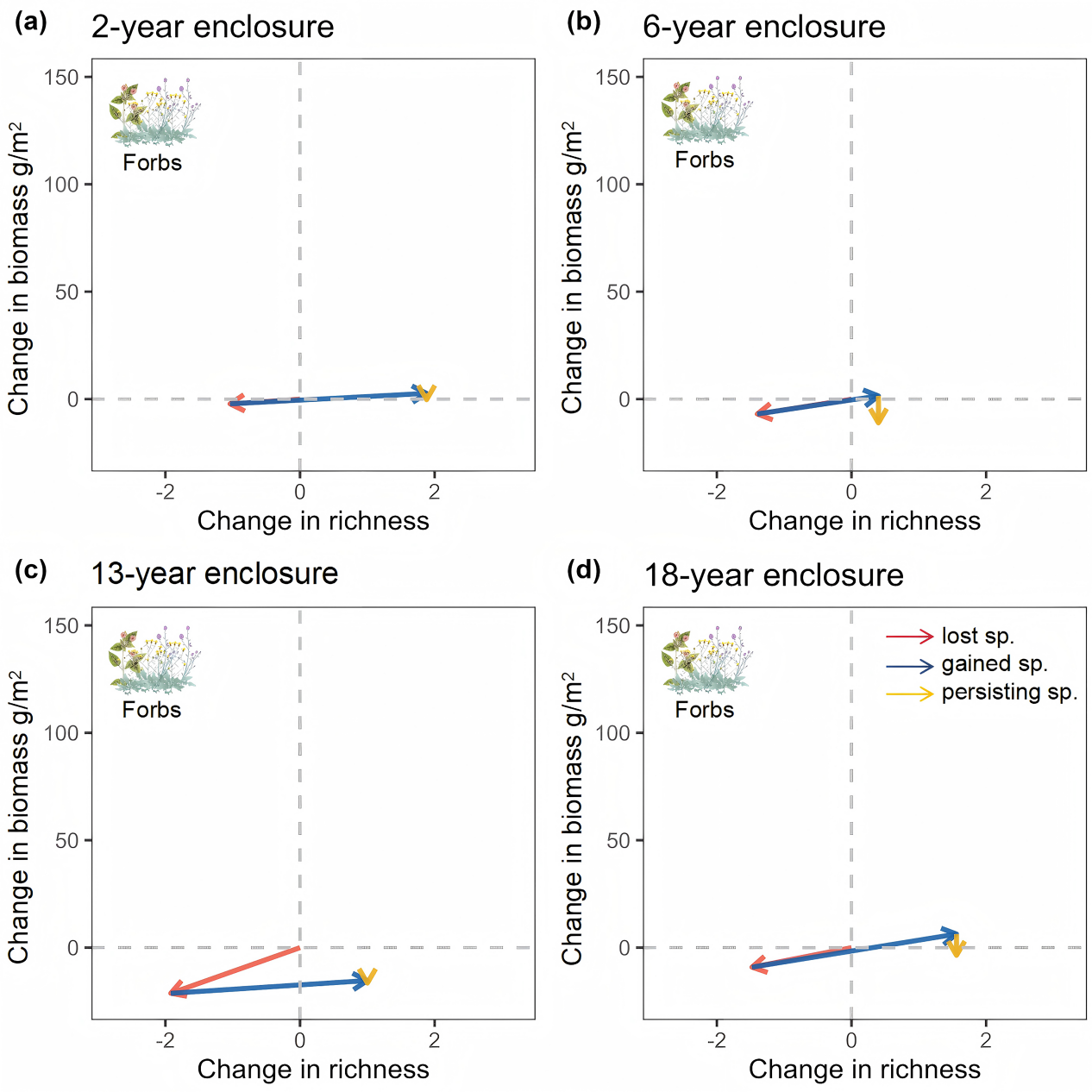


Figure S6 Changes in diversity and aboveground biomass of forb species under different enclosure durations.
